# Editorial: Computational approaches in pharmacological research and drug development: how to overcome the challenges of complex matrices in ethnopharmacology, therapeutics, and formulation design

**DOI:** 10.3389/fphar.2026.1912677

**Published:** 2026-07-22

**Authors:** Adnan Amin, Mozaniel Santana de Oliveira

**Affiliations:** 1 Department of Life Sciences, Yeungnam University, Gyeongsan, Republic of Korea; 2 Graduate Program in Pharmaceutical Sciences, Institute of Health Sciences, Federal University of Pará, Belém, Brazil

**Keywords:** chemical fingerprinting, computational approaches, ethnopharmacology, experimental pharmacology, pathway validation

## Introduction

Pharmacological research and drug development are being reshaped by the advancement of robust computational tools. Such modern tools allow for the precise prediction of drug targets, compound prioritization, pharmacokinetic analysis, formulation design, and optimization. Multiple stages of modern drug development use sophisticated virtual screening, molecular dynamics, network analysis, and artificial intelligence (Dara et al., 2022; Sadybekov and Katritch, 2023). The relevance of these modern tools is related to the fact that biologically active formulations generally comprise diverse metabolites rather than a single entity, thus making them appropriate for natural products and ethnopharmacology.

Despite such advancements, the pharmacological evaluation of metabolites cannot be simply reliant on computational prediction alone. This is based on the fact that medicinal plants and traditional medicines primarily comprise multiple metabolites from diverse classes and may develop synergistic, antagonistic, or potential metabolism-dependent effects. Furthermore, the biological activity of metabolites also depends on botanical origin, mode of extraction, route and dose of administration, and bioavailability. Thus, computational hypotheses must be supported by chemical characterization, experimental pharmacology, and, where possible, pharmacokinetic or clinical evidence (Vamathevan et al., 2019; Heinrich et al., 2020; Heinrich et al., 2022).

This Research Topic integrates computational approaches with pharmacological research and the drug development of complex natural matrices, their ethnopharmacological relevance, and formulation design. The studies comprising *in vitro, in vivo*, and clinical validation data integrated with computational prediction were mainly included, whereas purely *in silico* investigations without pharmacological confirmations were discouraged.

## From complex matrices to experimentally tested mechanisms

Two studies addressed metabolic diseases by combining computational prioritization with experimental validation. He et al. studied Yiqi Huoxue Yangyin Decoction, a five-botanical traditional formulation, in diabetic nephropathy. The authors defined its chemical profile using UPLC–MS/MS and integrated network pharmacology, transcriptomics, cellular studies, and a db/db mouse model. Their findings support a model in which the formulation attenuates podocyte injury by suppressing the METTL3-mediated m6A modification of mTOR and restoring autophagy. This work suggests the importance of linking the chemical identity of a complex preparation to pathway-level predictions and validation in cellular and disease models.

Feng et al. used database mining and ADME-based prioritization to identify peimisine from Fritillariae Cirrhosae Bulbus as a candidate for type 2 diabetes. Network and enrichment analyses were followed by glucose uptake and gluconeogenesis assays in HepG2 cells. Peimisine improved glucose utilization and suppressed gluconeogenic markers in the tested models, while HSP90AA1 emerged as a candidate target from network analysis and docking. Together, these studies show how computational approaches can narrow complex phytochemical spaces and generate plausible mechanisms, while emphasizing that predicted targets require direct target engagement and causal validation before being established.

## Clinical evidence and hypothesis-generating computation

Zeng et al. assessed Xiangdan Injection from a different translational perspective by placing clinical-use data first. Their retrospective analysis identified recurrent patterns of inappropriate administration, including unclear indications, unsuitable solvent selection, repeated medication, and prolonged treatment. Network pharmacology and docking were then used as exploratory tools to propose possible constituents, targets, and pathways relevant to pharmacological activity and safety. This separation between observed clinical practice and computational hypothesis generation is important. The retrospective data allow the identification of actionable medication-use risks, whereas the *in silico* findings provide questions for future experimental investigation rather than mechanistic proof. Thus, this study shows how computational analysis can complement, but should not override, evidence derived from real-world clinical use.

## Natural product pharmacology and rational mixture design

The remaining contributions showed two complementary strategies for advancing natural product therapeutics. The investigation by Ali et al. assessed folenolide using *in vivo* models of pain, inflammation, fever, and acute toxicity, together with docking against selected pharmacological targets. Folenolide exhibited analgesic, anti-inflammatory, and antipyretic activities in mice without signs of acute toxicity under the tested conditions. Docking provided possible molecular explanations, but the experimentally observed phenotypes remained the prime evidence, and additional biochemical target validation is needed.

Li et al. addressed the challenge of optimizing a defined multicomponent system derived from *Salvia miltiorrhiza* and *Dalbergia odorifera*. Using an improved simplex-centroid mixture design, the authors modeled combinations of salvianolic acid B, tanshinone IIA, butein, and formononetin and selected an optimized proportion through a multi-objective analysis. This was followed by an evaluation using oxygen glucose-deprived cardiac cell models and mice with myocardial infarction. This investigation is an interesting example of using computational pharmacology for an extensive workout, including the optimization of component ratios before experimental confirmation, instead of simple prediction. Both of these studies significantly explained the standing of computational tools in pharmacological research, particularly in the context of rationalizing the interactions among multiple known components using a statistical design.

## Outlook

Thus, a consistent message appears after a careful assessment of all published studies, i.e., computational assessments are most informative only when tailored to a defined workflow based on sound chemical and biological grounds. The botanical authentication, standard extraction, detailed chemical profiling, and chemo-taxonomical markers are therefore needed for designing complex ethnopharmacological matrices. It has also been emphasized that computational results are only taken according to their evidential level. For instance, molecular docking analysis predicts the compatibility of structures and interactions, network centrality ranks nodes and enrichment analysis classifies associations. It is therefore evident that none of these tools alone can establish the clinical efficacy or a molecular mechanism of the analyzed metabolites.

Thus, further research should focus on fortifying this evidence chain using direct target-engagement analysis, the perturbation of predicted pathways, pharmacokinetic, metabolite profiling, and potential clinical evaluation if needed. In formulation and mixture research, predicted values must be aligned with measurable exposure, stability, therapeutic performance, and toxicity. By integrating chemical rigor, computational prioritization, experimental pharmacology, and translational context, this Research Topic provides a practical framework for converting complex natural matrices into reproducible and clinically relevant therapeutic hypotheses.
